# Amino acids chemical stability submitted to solid state irradiation: the case study of leucine, isoleucine and valine

**DOI:** 10.1186/s40064-015-1332-9

**Published:** 2015-09-22

**Authors:** Cristina Cherubini, Ornella Ursini

**Affiliations:** Institute of Chemical Methodologies-National Research Council of Italy, Via Salaria Km. 29,300, 00015 Monterotondo, RM Italy

**Keywords:** Gamma radiation, Meteoritic context, Radioracemization, Leucine, Isoleucine, Valine

## Abstract

A solid state radiolysis was conducted to examine the reactivity of amino acids towards high energy dose of gamma radiations. The presence of amino acids in the bulk of meteorites has raised the question of “if” and eventually “how” they could have been important in the development of life on Earth. The presence of radioactive elements in Solar System bodies could have played a crucial role in amino acids survival and in the formation of different organic molecules. The radioactive elements produced a total radiation dose of 14 MGy during the life of Solar System (4.6 × 10^9^ years). The aim of this study is to investigate the amino acids capacity to survive at a dose of γ-irradiation equivalent to 1.05 × 10^9^ years of Solar System life. In particular, we examined the behavior of three essential amino acids (leucine, isoleucine and valine) submitted to a total dose of 3.2 MGy. We choose to irradiate l-enantiomers to analyze the behavior of a single enantiomer to radiations. We identified the radiation products formed in solid state radiolysis by mass spectrometric analysis and we were able to enlighten some common reactions. These reactions are particularly important to rationalize the formation of prebiotic molecules. Moreover, we studied the radioracemization process, the formation of d-enantiomer promoted by γ-irradiation.

**Electronic supplementary material:**

The online version of this article (doi:10.1186/s40064-015-1332-9) contains supplementary material, which is available to authorized users.

## Background

Even before the discovery of complex organic matter on Solar System bodies, many researchers had hypothesized a significant influence of extraterrestrial material for the development of life on Earth (Orò 1961; Urey 1962). Essentially there are two theories on how life on Earth has originated. For the first theory, life on Earth has an endogenous abiogenic origin. Therefore, the primitive Earth conditions have promoted the formation of organic molecules from simple molecules. This theory is confirmed by Miller-Urey experiments (Miller 1953) in which small molecules such as H_2_O, CH_4_, NH_3_ and H_2_, lead to the formation of several amino acids. According to the second theory, life on Earth has developed thanks to an exogenous input from extraterrestrial material. This theory asserts that an important role in evolution of life is played by extraterrestrial prebiotic molecules, arrived on Earth through meteorites delivery (Orò 1961; Chyba et al. 1990; Berstein et al. 1999).

From the first studies conducted on small Solar System bodies, it was evident the presence of different organic molecules (Kvenvolden et al. 1970). The carbonaceous chondrides are particularly rich in organics, amino acids among them. These organic molecules were submitted to different high energy irradiation processes. In extraterrestrial environment different kind of energy sources are universally present (proton, electrons and photon beam, gamma ray or UV) and they could be considered the promoters for the abiogenic synthesis of biomolecules in space (Kobayashi et al. 2001). The discovery of the amino acids existence in meteorites and other Solar System bodies (Cronin and Pizzarello 1983; Cronin et al. 1988; Cronin and Chang 1993) was the beginning of massive studies aimed to explain how they were formed and how they were able to survive in a meteoritic environment (Kobayashi et al. 1998; Ehrenfreund and Charnley 2000; Berstein et al. 2002; Munoz Caro et al. 2002; Takano et al. 2004).

Amino acids found in meteorites are the result of two types of phenomena. Firstly, they are preserved from cosmic rays action by their incorporation in the meteoritic bodies at a depth >20 m. Secondly, the radionuclide decay could be the main cause of amino acids degradation inside the meteoritic body (Draganic et al. 1993). Different radioactive elements were detected in meteorites and other Solar System bodies (Anders and Grevesse 1989). According to Urey’s works (Urey 1955, 1956) radioactive elements, principally ^40^K, ^232^Th, ^235^U, ^238^U and ^26^Al, produced a total radiation of ≈14 MGy during the life of Solar System (4.6 × 10^9^ years). The mere presence of ^26^Al is responsible of producing ≈11 MGy in the first billion years of existence of the Solar System (Kohman 1997). The effect of radiations is particularly relevant due to the fact that amino acids were discovered in a little enantiomeric excess (Cronin and Pizzarello 1997, 1999; Pizzarello and Cronin 2000). This relevant finding induced the proposal of some theories about the evolution of life on Earth. The most accepted one involved chiral amplification mechanism of prebiotic molecules delivered in enantiomeric excess by meteorites: from the little enantiomeric excess of amino acids and sugars, biological molecules developed selecting l-amino acids and d-sugar (Podlech 2001; Pizzarello 2004).

It is well known (Cataldo et al. 2008) that a chiral molecule submitted to ionizing radiations can experience radioracemization, a degradation process leading to a reduction in optical activity as a consequence of two main events: firstly, the radiolysis of chiral molecules and their degradation into products without asymmetric centre; secondly, the inversion of chiral centre due to the high energy involved during irradiation.

To study the effect of radiations on solid state amino acids, we have already carried out some experiments (Cataldo et al. 2011a, b, 2013a, b; Iglesias-Groth et al. 2011). These studies were conducted on both proteinaceous and non proteinaceous amino acids, the latter ones present in higher amount in meteorites.

The aim of the present study is to investigate the stability of three amino acids (valine, isoleucine and leucine) irradiated in the solid state at 3.2 MGy, without oxygen and humidity. These three amino acids are considered to be primitive amino acids, in other word amino acids formed at the first stages of Solar System life (Pascal et al. 2005**).** We choose to irradiate l-enantiomers to analyze the behavior of a single enantiomer to radiations. We tested the stability of three amino acids submitted to solid state irradiation, identifying the radiation products and investigating the amino acids ability to retain their initial signature.

## Experimental

**Fig. 1:**
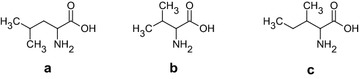
Structure of analyzed amino acids: leucine (**a**), valine (**b**) and isoleucine (**c**)

The amino acids l-valine, l-leucine and l-isoleucine (Fig. 1) were obtained from Sigma-Aldrich (Milan, Italy) and used as received (reagent grade min 98 % TLC).

### Irradiation experiments

The irradiation with γ rays was conducted in solid phase through a ^60^Co Gammacell 220 from Atomic Energy of Canada. The dose rate was 0.8 kGy/h for a total dose of 3.2 MGy. Each amino acid was irradiated, in absence of air, separately from one another.

The amino acids were weighted (300 mg each) and introduced into a Durhan borosilicate glass vial as pure solid. The vacuum was obtained using a high vacuum line. After having pumped off the air, the glass vial was sealed at the flame.

### Mass spectrometric analysis

The amino acids were analyzed by a mass spectrometer Finnigan LXQ, a linear ion trap system equipped with an ESI ion source. The mass spectra were obtained by a direct injection of amino acids solutions in the mass spectrometer. l-Val, l-Leu and l-Ile were dissolved in 1 mL methanol and 1 mL of ammonium acetate solution 45 mM. The concentration for the three amino acids were, respectively, 1.0 × 10^−2^ M, 7.8 × 10^−3^ M and 6.7 × 10^−3^ M and they were the same for irradiated and pristine samples, in order to have a more direct comparison for every amino acid. The flow injection was 10 µL/min and the analysis were conducted both in positive and in negative ion polarity mode, so as to identify products which retain the basic group (positive ion mode) and products which maintain the carboxylic group (negative ion mode). New products originated during irradiation were discovered comparing the mass spectra of pristine, non radiolyzed amino acids and the spectra of samples irradiated in absence of air.

Whenever a new ion was detected in the spectrum, it was isolated in the ion trap and fragmented by collision induced dissociation (CID), operating a MS^n^ analysis, with n up to 5. The possibility to isolate and fragment the ions derived from a first CID analysis enabled us to determine the chemical structure of irradiation products, drawing a fragmentation pathway for every new ion.

Coupling the ESI–MS with a HPLC system equipped with a teicoplanine based chiral column allowed us to measure the amount of d-enantiomer formed by irradiation process. The HPLC analysis was made using a Shimadzu liquid chromatograph LC-10AD VP. The chiral column was a stainless steel column, Astec CHIROBIOTIC™ T, 5 µm particle size, 150 mm × 4.6 mm. The mobile phase was a mixture of methanol, water and formic acid (70 CH_3_OH—30 H_2_O—0.02 CH_2_O_2_) and the flow rate was 0.8 mL/min, splitted with a ratio 60/40 (waste/mass spectrometer ESI source). The purpose of the splitting was to increase sensitivity of mass spectrometer without losing resolution from the chiral column. A calibration curve was constructed with standard solution of l- and d-enantiomer at different concentration (2, 5, 10, 15 and 20 % of d-enantiomer solution) to estimate the amount of d-enantiomer formed. From the area data, provided by the Xcalibur program, it was possible to calculate the amount of d-enantiomer.

## Results

### Identification of radiation products of l-Leucine (l-Leu)

**Fig. 2:**
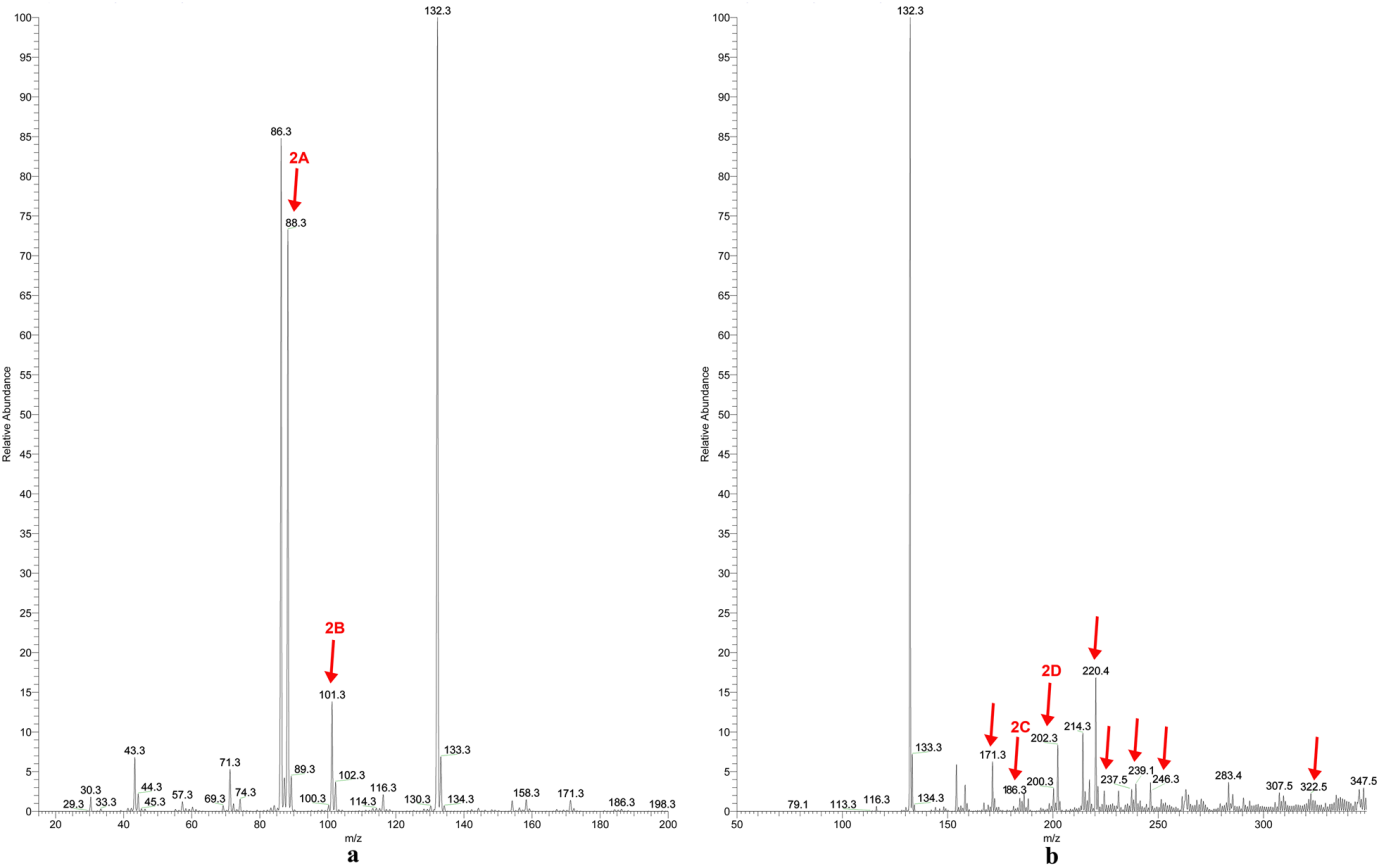
Mass spectra of l-Leu irradiated under vacuum condition in positive ion mode at low mass range (*m*/*z* 15–200) (**a**) and normal mass range (*m*/*z* 50–800) (**b**). The irradiated l-Leu was dissolved into a solution 1:1 of MeOH-AcNH_4_ 45 mM to obtain a concentration of 7.8 mM. The mass spectra for l-Leu standard are reported in the Additional file 1

The mass spectra at different mass range of l-Leu irradiated in absence of air are reported in Fig. 2a, b. The spectra were recorded at low mass range (*m*/*z* 15–200, Fig. 2a) and at normal mass range (*m*/*z* 50–800, Fig. 2b), both in positive ion mode. The ions derived from radiation products are enlighten by an arrow.

**Fig. 3:**
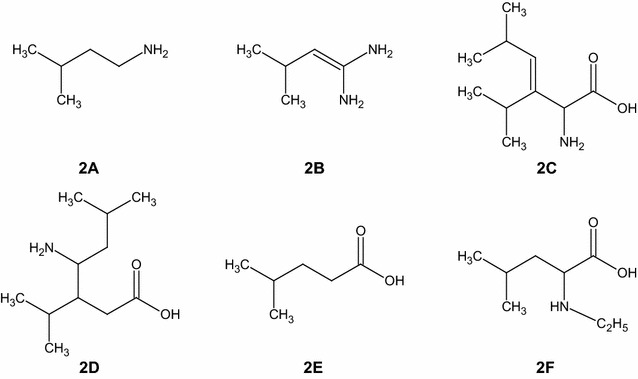
Hypothesized neutral structures corresponding the ions *m*/*z* 88 (**2A**), *m*/*z* 101 (**2B**), *m*/*z* 186 (**2C**), *m*/*z* 202 (**2D**), *m*/*z* 115 (**2E**) and *m*/*z* 158 (**2F**). The fragmentation mass spectra for these ions are reported in the Additional file 1

The ions *m*/*z* 132 (protonated leucine), *m*/*z* 154 (Na^+^ leucine adduct) and *m*/*z* 263 (leucine proton bound dimer) were already present in the pristine sample of l-Leu. The radiolysis products detected in the normal mass range are consequences of radicals assembling processes. In fact, the amino acids were wrecked into radicals due to the high energy developed in the radiolysis process. These radicals remained in the vials during the irradiation process. In this lapse of time they could react via different pathways: react with each other, react with neutral molecules or rearrange. The interaction with another radical or a neutral molecule led to the formation of products with a higher molecular mass than the amino acid itself. Not for all the ions it was possible to determine the molecular structure because it was not easy to isolate ions with low abundances. The MS isolation procedure is the first necessary step to proceed with CID analysis, essential to elucidate the structures of the ions. Some of the hypothesized structures of the radiolysis products are shown in the Fig. 3. The structures reported refer to ions detected in positive ion mode and low mass range (**2A**, **2B**), positive ion mode and normal mass range (**2C**, **2D**), negative ion mode and normal mass range (**2E**, **2F**).

The possibility to make a mass spectrum at a lower mass range was important to observe the presence of other radiolysis products, as the one derived from decarboxylation process (**2A**). The decarboxylation product 3-methylbutylammine (**2A**) is the precursor of the product observed as the ion *m*/*z* 101, corresponding to 1-amino-3-methylbut-1-enylamine (**2B**). It results from the reaction of **2A** ammine with an ammonia radical. Besides the dual ESI ion polarity (positive and negative) mode provided several important indications. The mass spectrum in negative ion mode showed the presence of other radiolysis products, first of all the product of deamination (**2E**). Some of the ions in negative ion mode were also present if the spectrum was recorded in positive ion mode, as positive ion. It is plausible to suppose that if a product is visible both in negative and positive ion mode, the amino and carboxylic groups are kept in the radiation product. Whenever a ion is present only in one of the two ionization mode, it means that one of the functional group is not present in the molecule or it has an impediment to the ionization. This is the example of the ion *m*/*z* 158 (**2F**) visible in negative ion mode, normal mass range, where the ethyl group is thought to be bound at the amino group. The consequent formation of a secondary amino group R-NH-R^1^ results in no detection in positive ion mode.

### Identification of radiation products of l-Isoleucine (l-Ile)

**Fig. 4:**
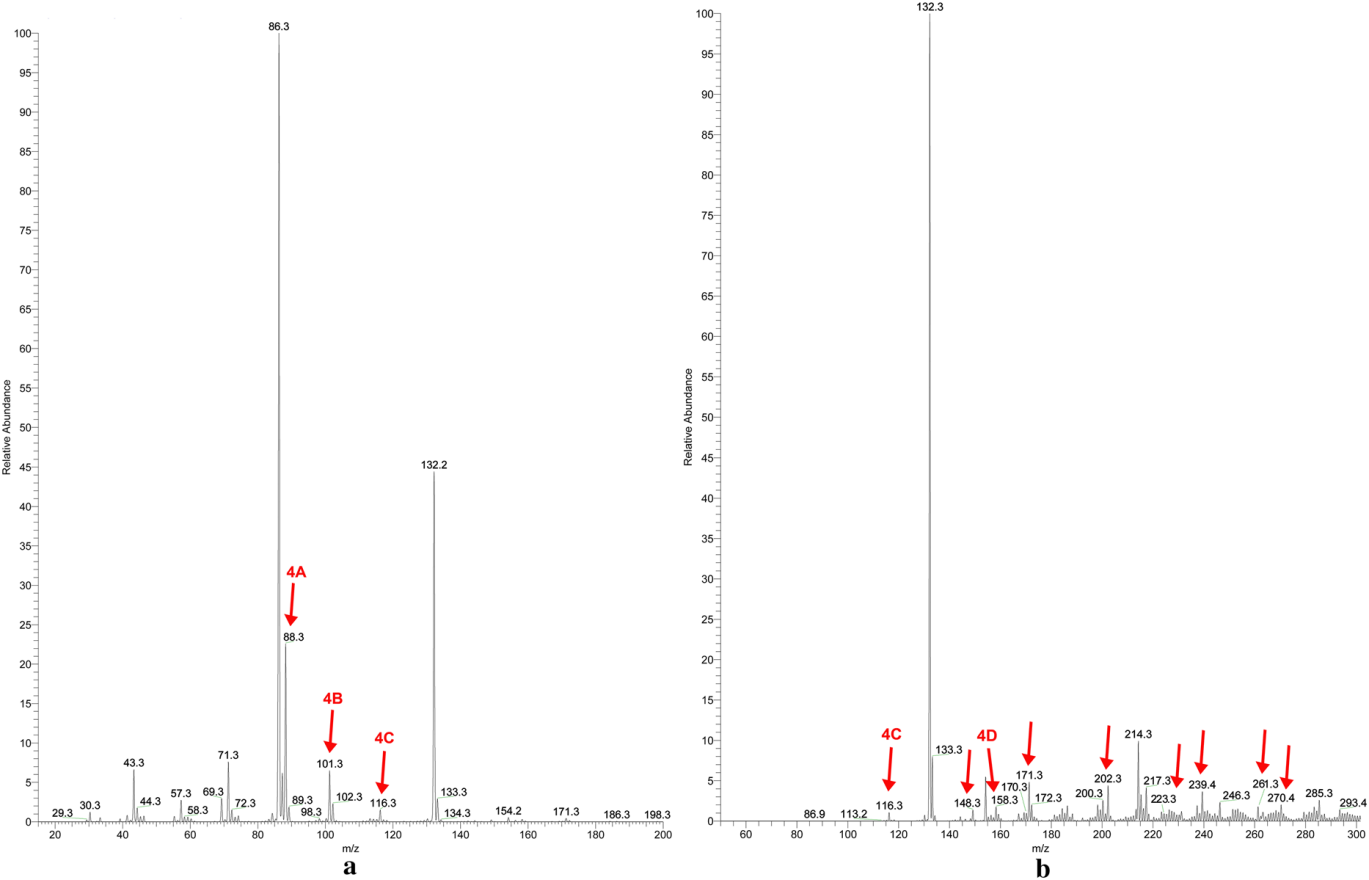
Mass spectra of isoleucine irradiated in absence of air in positive ion mode, at low mass range (**a**) and at normal mass range (**b**). The concentration for the samples was 6.7 mM in a solution 1:1 MeOH-AcNH_4_ 45 mM. The mass spectra for l-Ile standard are reported in the Additional file 1

In Fig. 4a, b are reported the mass spectra of l-Ile irradiated in absence of air, recorded in positive ion mode, at low mass range (*m*/*z* 15–200; Fig. 4a) and at normal mass range (*m*/*z* 50–800; Fig. 4b). The ions derived from radiation products are enlighten by an arrow.

Contrary to what happens to leucine, in isoleucine case the ions that reveal the formation of a oxidation product were detectable in vacuum irradiation experiment. A particular isoleucine irradiation product is observed as ion *m*/*z* 148. It reveals the formation of an oxidation product not identified neither for leucine nor for valine in vacuum irradiation experiment. The ion *m*/*z* 148 detected in positive ion mode and ion *m*/*z* 146 detected in negative ion mode, led us to suppose a small presence of humidity in the vial. However, even if the oxidation product was noticed, it had a very low percentage (0.2 %).

**Fig. 5:**
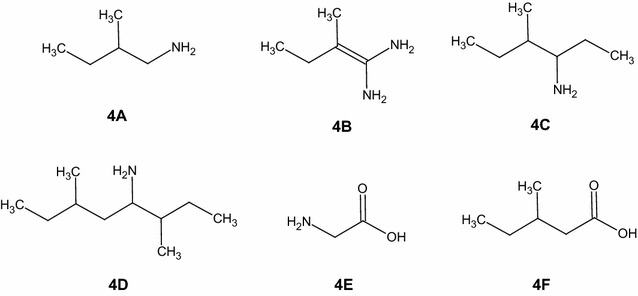
Hypothesized neutral structures of the ions *m*/*z* 88 low mass positive (**4A**), *m*/*z* 101 low mass positive (**4B**), *m*/*z* 116 low and normal mass positive (**4C**), *m*/*z* 158 normal mass positive (**4D**), *m*/*z* 74 low mass negative (**4E**), *m*/*z* 115 norm mass negative (**4F**). The fragmentation mass spectra for these ions are reported in the Additional file 1

As it happens for leucine with the formation of 1-amino-3-methylbut-1-enylamine, in the same way the product of decarboxylation of isoleucine (**4A**) can react with an ammonia radical and lead to the formation of 1-amino-2-methylbut-1-enylamine (**4B**). Due to the absence of the carboxylic group, the ion *m*/*z* 101 (**4B**) was detected only as a protonated ion, while the deamination product (ion *m*/*z* 115, **4F**) could only be observed as a negative ion. The ion *m*/*z* 74 (**4E**) was identified as glycine ion and it was detected in the negative ion mode, low mass range. The impossibility to isolate and fragment some ions due their low relative abundances prevented us to identify all the products, although it was possible to obtain quantitative data. (see “Quantitative data”). The remaining ions could be identified as products of assembling radical reactions occurred in the vial during the long time of irradiation (Fig. 5).

### Identification of radiation products of l-Valine (l-Val)

**Fig. 6:**
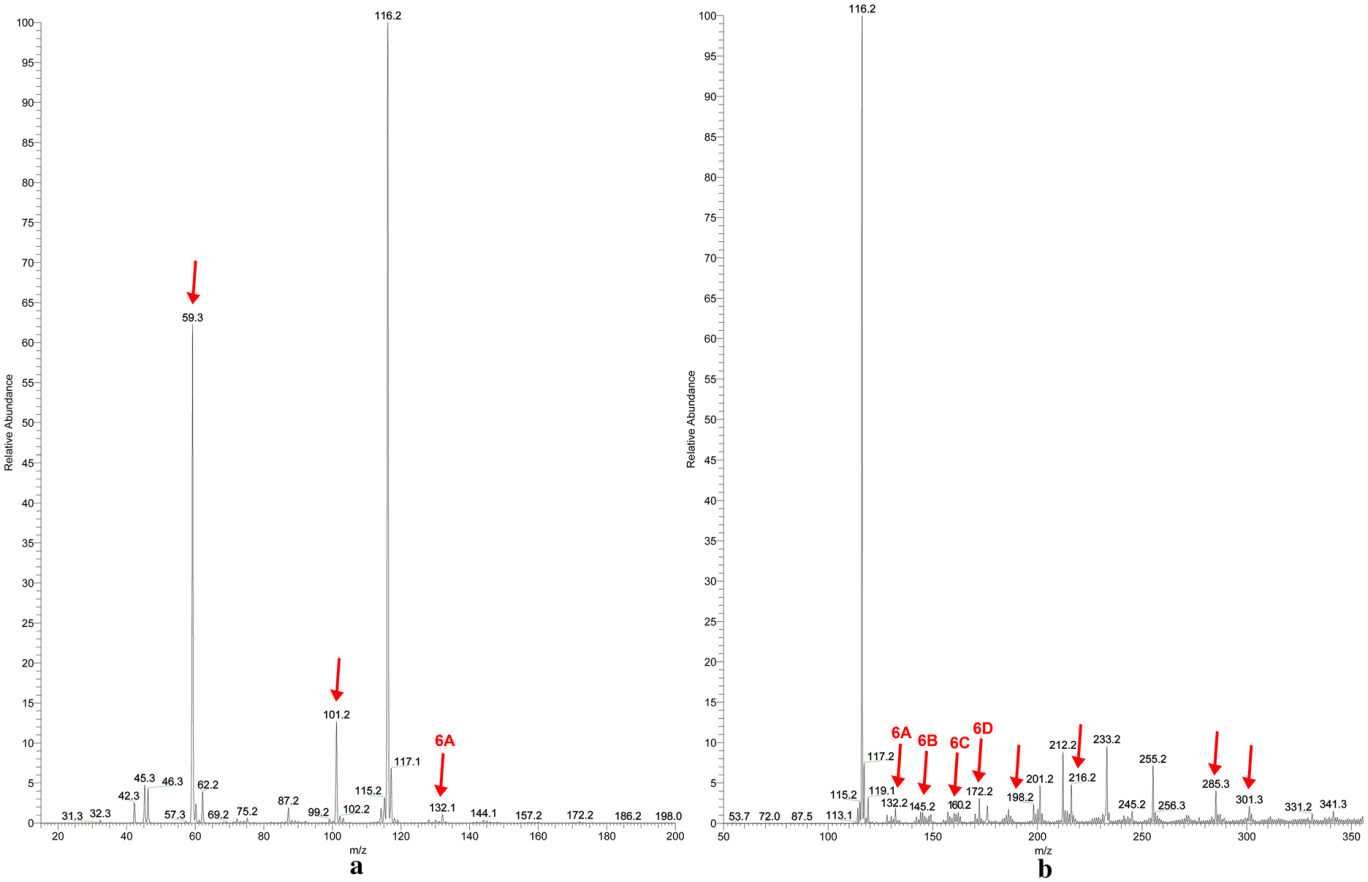
Mass spectra of valine irradiated in absence of air at low mass range (**a**) and at normal mass range (**b**). The spectra were recorded in negative ion mode. The concentration was 10 mM in a solution 1:1 MeOH-AcNH_4_. The mass spectra for l-Val standard are reported in the Additional file 1

The mass spectra of l-Val irradiated in absence of air is reported in Fig. 6a, b. In Fig. 6a is reported the spectrum at low mass range (*m*/*z* 15–200), while in Fig. 6b the spectrum at normal mass range (*m*/*z* 50–800), both recorded in negative ion mode, where the majority of radiation products are detected. The ions derived from radiation products are enlighten by an arrow.

The mass spectra of irradiated l-Val showed a variety of ions correlated to radiation products. Other than decarboxylation product (ion *m*/*z* 74) and deamination product (ion *m*/*z* 101), some interesting ions were detected, mainly in negative ion mode.

**Fig. 7:**
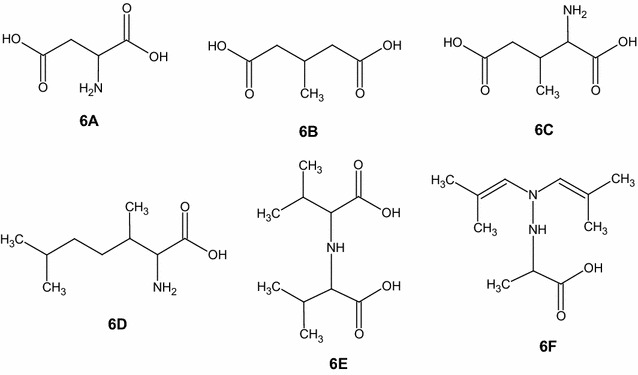
Some hypothesized neutral structures of the ion *m*/*z* 132 normal mass negative (**6A**), ion *m*/*z* 145 normal mass negative (**6B**), ion *m*/*z* 160 normal mass negative (**6C**), ion *m*/*z* 174 normal mass positive (**6D**), ion *m*/*z* 216 normal mass negative (**6E**), ion 213 normal mass positive (**6F**). The fragmentation mass spectra for these ions are reported in the Additional file 1

The ions *m*/*z* 132 (**6A**), 145 (**6B**) and 160 (**6C**) had all a double carboxylic group (Fig. 7). They were the direct consequence of the decarboxylation induced by radiation and the resulting presence of CO_2_ in the vial during irradiation. In the same way, the radicals derived from the valine side chain could react with a neutral valine molecule and generate a series of products which were represented by the ions *m*/*z* 174 (**6D**), 198 and 285. Decarboxylation and deamination products could create other molecules identified in the mass spectra as the ions *m*/*z* 216 (**6E**) and 213 (**6F**) (Fig. 7).

### Quantitative data

Taking into account the mass data, it was possible to estimate the amount of radiation products. In this way we were able to establish not only the reactivity of amino acids submitted to radiations, but also to evaluate their capacity to survive in the body of meteorites at the same radiation dose. The relative percentage of the radiolysis product ions are reported in the Table 1.

**Table 1:** Relative percentage of radiation products detected in the mass spectra of l-Leu, l-Ile and l-Val irradiated in vacuum condition

Amino acid	Loss of small radicals	%	Addition of radicals to amino acids	%	Addition of radicals to primary radiation products	%
Leucine	*m*/*z* 88^a,b^	8.7	*m*/*z* 158^e^	0.1	*m*/*z* 101^b^	1.7
	*m*/*z* 115^e^	1.4	*m*/*z* 175^e^	3.8	*m*/*z* 171^c^	1.8
			*m*/*z* 186^c^	0.6	*m*/*z* 202^c^	0.8
					*m*/*z* 220^c^	0.5
					*m*/*z* 237^c^	4.6
					*m*/*z* 239^c^	0.1
					*m*/*z* 244^e^	1.1
					*m*/*z* 322^c^	1.2
					*m*/*z* 446^e^	0.1
		10.1		4.5		11.9
Isoleucine	*m*/*z* 74^d^	0.1	*m*/*z* 229^e^	0.5	*m*/*z* 101^b^	0.4
	*m*/*z* 88^a,b^	4.2	*m*/*z* 244^e^	1.0	*m*/*z* 116^b^	0.3
	*m*/*z* 101^e^	0.9	*m*/*z* 343^e^	0.6	*m*/*z* 158^c^	0.4
	*m*/*z* 115^e^	4.8			*m*/*z* 170^c^	0.2
					*m*/*z* 171^c^	1.0
					*m*/*z* 202^c^	0.9
					*m*/*z* 223^c^	0.8
					*m*/*z* 239^c^	0.6
		10.0		2.1		4.6
Valine	*m*/*z* 59^d^	6.3	*m*/*z* 160^e^	0.3	*m*/*z* 87^b^	2.3
	*m*/*z* 74^b^	3.6	*m*/*z* 174^c^	0.7	*m*/*z* 88^b^	0.8
	*m*/*z* 101^d^	1.9	*m*/*z* 198^e^	0.5	*m*/*z* 132^e^	0.3
			*m*/*z*z 216^e^	0.7	*m*/*z* 145^e^	0.2
			*m*/*z* 285^e^	0.8	*m*/*z* 213^c^	0.9
					*m*/*z* 307^c^	0.8
		11.8		3.0		5.3

^a^The percentage is the sum of ion *m*/*z* 88 and its fragment *m*/*z* 71, derived from ESI ionization

^b^Positive ion mode, low mass range

^c^Positive ion mode, normal mass range

^d^Negative ion mode, low mass range

^e^Negative ion mode, normal mass range

In Table 1 are reported the three categories of products formed during irradiation experiments. For every product is shown a relative percentage calculated using the Xcalibur program of the mass spectrometer and taking into account the quantity of amino acid preserved during irradiation. The loss of small radicals from the amino acid molecules is the first category. It is represented by all the products in which there is the loss of a small group, such as CO_2_, NH_3_, CH_4_. Decarboxylation and deamination are the most important irradiation processes. They are considered to be the starting point of subsequent radiation pathways, due to the fact that the formed primary radiation products were able to further react. In the fourth column are reported the ions derived from an addiction of radicals to the amino acid molecules. These radicals could be either small (CO_2_, NH_3_, CH_4_) or large radicals, the latter derived from the amino acid side chain. The remaining column refers to the products of small and large radicals addition to those primary radiation products already mentioned. The mechanism of formation of these products will be discussed in the following sections.

## Discussion

**Fig. 8:**
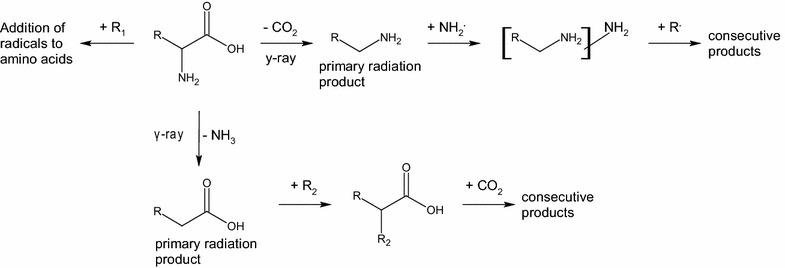
Processes induced by radiation, starting from decarboxylation and deamination of amino acid neutral molecules

Due to the high energy involved in the radiation process, a series of reactions could occur in the vial, increased by the long time of irradiation. Studying amino acids behavior towards radiation with mass spectrometry, it was possible to delineate some common mechanisms of reaction. Deamination and decarboxylation are well known reactions induced by radiations (Bonifačić et al. 1998; Nordén et al. 1985) and were easily observed for each amino acid (Fig. 8). The deamination products were detected in negative ion mode, while the decarboxylation products in positive ion mode.

In a similar way the radiations could cause losses of small molecules from amino acids side chain, leading to products different for each amino acid. These small molecules, comprehensive of ammonia and carbon dioxide, remained in the vial during irradiation and reacted mainly with the primary radiation products.

The primary radiation products reacted easily with radicals, either small (CO_2_, NH_3_, CH_4_) or large (from the amino acid side chain) radicals. It is thought that these radicals remained closely to the primary radiation products due to the fact that both of them were directly produced, at the same time, by irradiation processes. These type of products are significant in terms of relative quantity as reported in Table 1.

The radicals could also virtually react with neutral amino acids, present in great abundance. However, the additions of radicals to amino acids are weakly detected. The lower quantity of these products can be justified by the fast reaction between radicals and primary radiation products due to their proximity, as daughters of the same amino acid molecule.

Particularly important is the reaction in which there was a substitution of an amino hydrogen with an alchilic group (product **2F** for leucine; products **6E** and **6F** for valine). The substitution of an amino hydrogen is easily recognizable by the fragmentation spectra of the isolated ion. In fact, whenever the ammonia group is substituted there is not the typical fragment ion of ammonia loss (see Additional file 1). Moreover, if there is an impediment to the protonation of ammonia group, the product is not detectable in positive ion mode. The alchilic substitution of ammonia group is particularly interesting in a meteoritic context due to the presence of *N*-substituted amino acids (Cronin and Pizzarello 1983). We believe that radiations took actively part in the formation of *N*-substituted amino acids.

What it is important considering the radiations is the role they played in the survival of l-enantiomer. It is well known that radiations can reduce the optical activity promoting radioracemization (Cataldo et al. 2008). The chromatographic analysis (see Additional file 1) allowed us to identify the d-enantiomer formed as a result of radioracemization process during irradiation. It is necessary to pay attention to isoleucine, one on the two proteinogenic amino acid, together with threonine, which has two chiral centre (α and β carbon). Due to the presence of these two chiral centre, isoleucine can exist as four stereoisomers which can be seen as two pairs of enantiomers: l- and d-isoleucine, l- and d-allo-isoleucine. The simultaneous inversion of the two chiral centre is unlikely and consequently the formation of d-Ile (Bada and Schroeder 1972). The most common interconversion is the α epimerization which involves the formation of d-allo-isoleucine. In fact, the α carbon can react easily thanks to electron-withdrawing and resonance stabilization effects. On the contrary the β carbon is protected from the amino and carboxylic groups and the β epimerization is a slow rate process.

We calculate the radiolytic formed d-enantiomer integrating individual chromatographic areas of l- and d-enantiomer. In Table 2 we report the amount of d-enantiomer formation either in presence of air and in vacuum condition.

**Table 2:** Percentage of d-enantiomer formation in presence or absence of air

Amino acid	% of d-enantiomer formation in presence of air	% of d-enantiomer formation in vacuum condition
Valine	0.6	1.0
Isoleucine	0.3	0.6
Leucine	n.d.	1.8

**Fig. 9:**
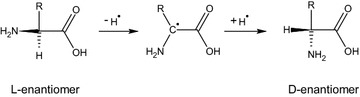
Scheme of the racemization induced by radiations

The presence of oxygen seemed to inhibit, at some extent, the formation of d-enantiomer. For l-Val and l-Ile the presence of d-enantiomer was halved by the oxygen action, while in the case of l-Leu there was no evidence of radioracemization when the irradiation occurred in atmospheric condition. The mechanism of radioracemization is established to be an homolytic cleavage of Cα-H bond (Neuberger 1948) (Fig. 9).

In presence of air, the radical H^·^ remains in close proximity of its breaking point and the racemization process is less probable. When the air is not present, the radical H^·^ became able to move and can easily promote a configuration inversion. In regard to the formation of the d-enantiomer, it is possible to observe a different behavior for the three amino acids. Considering the leucine, the formation of the d-enantiomer is greater in vacuum condition irradiation rather than in presence of air. This behavior could be rationalized taking into account the less steric hindrance of leucine Cα-H bond. The consequence is a easier homolytic cleavage of Cα-H bond, resulting in a more pronounced d-enantiomer formation when the irradiation is carried out in vacuum condition. On the other hand, in presence of air the leucine radical is ready to respond to other radicals produced by radiations. This fast reaction causes the formation of different molecules. In fact, as reported in Table 1, the products formed by consecutive radical additions are more pronounced respect to the other two type of products.

## Conclusion

The mass spectrometric study of irradiated amino acids in solid phase can give us some answers to the question of “how” and “how much” the amino acids were able to survive to radiations emitted by radioactive elements present in meteorites. The identification of radiation products can be achieved thanks to MS^n^ analysis, through isolation and fragmentation processes and CID technique. At the same time, a quantitative study is conducted to estimate the percentage of amino acids unmodified after irradiation. It seems that amino acids are relatively stable to radiations: despite the high radiation dose, all of the three amino acids survived at a percentage major to 70. This result is in agreement to what is found in meteorites, where leucine, isoleucine and valine are present in relative great amount (Cronin and Pizzarello 1983). Some amino acids are present in enantiomeric excess, with the l-enantiomer prevalent. It is thought that this excess was more relevant at initial phase of Solar System life and it had decreased due the process of racemization induced by radiation. The observation of radioracemization in solid state confirm this theory and open the possibility to further study this phenomenon.

## Additional file

**Additional file 1.** In the Supplemental Material Section the mass spectra of pristine (non irradiated) amino acids, the fragmentation mass spectra of irradiation products and the chromatograms of three irradiated amino acids are presented.
