# Supplementary material for: Amino acids chemical stability submitted to solid state irradiation: the case study of leucine, isoleucine and valine
Source: Springerplus. 2015 Sep 22;4:541. doi: 10.1186/s40064-015-1332-9 (PMC4579194; doi:10.1186/s40064-015-1332-9)
Supplement: Supplementary file 1 — Additional file 1. In the Supplemental Material Section the mass spectra of pristine (non irradiated) amino acids, the fragmentation mass spectra of irradiation products and the chromatograms of three irradiated amino acids are presented. [file 40064_2015_1332_MOESM1_ESM.docx]

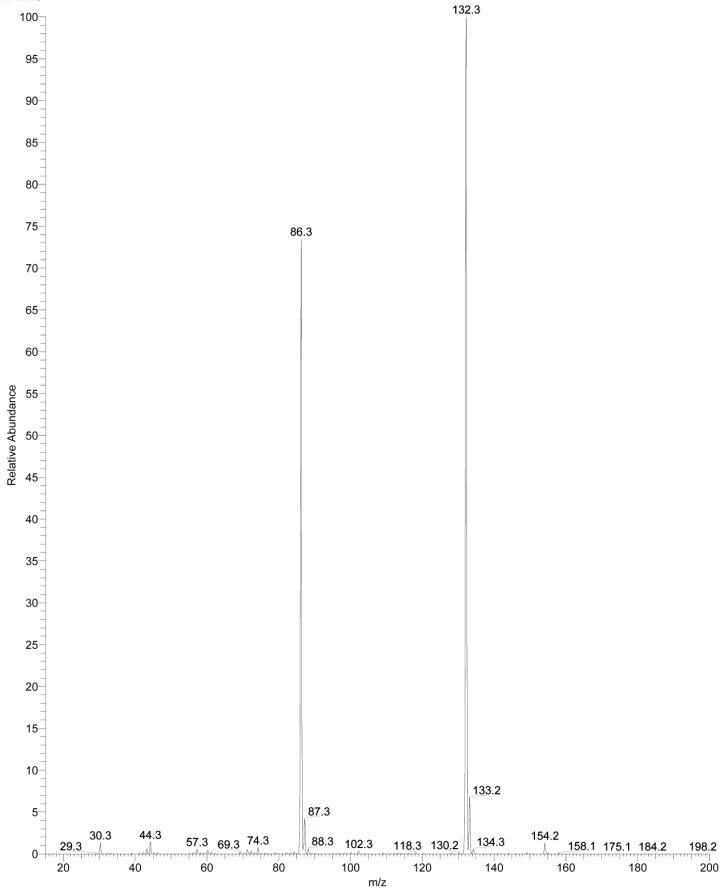


Mass spectrum of L-Leu standard in positive ion mode at low mass range (m/z 15-200). The standard L-Leu was dissolved into a solution 1:1 of MeOH-AcNH_4_ 45 mM to obtain a concentration of 7.8 mM


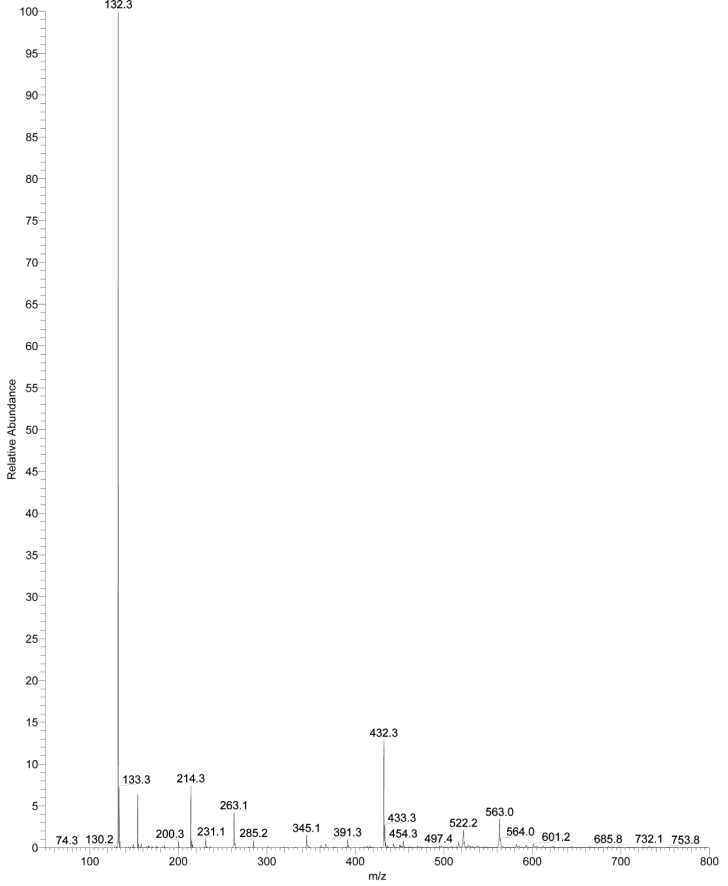


Mass spectrum of L-Leu standard in positive ion mode at normal mass range (m/z 50-800). The standard L-Leu was dissolved into a solution 1:1 of MeOH-AcNH_4_ 45 mM to obtain a concentration of 7.8 mM


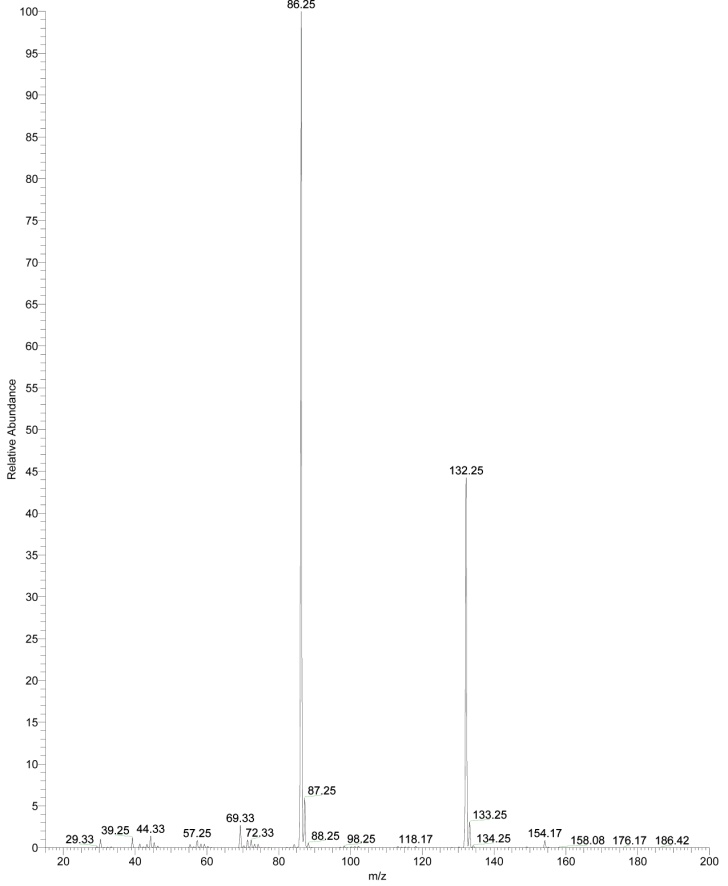


Mass spectrum of L-Ile in positive ion mode at low mass range (m/z 15-200). The concentration for the samples was 6.7 mM in a solution 1:1 MeOH-AcNH_4_ 45 mM


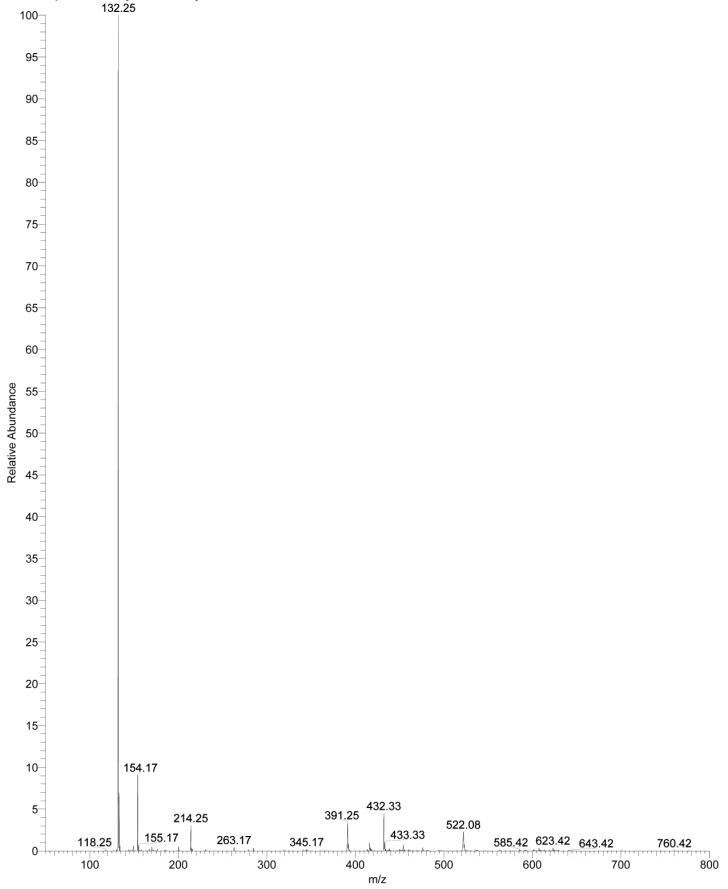


Mass spectrum of L-Ile in positive ion mode at normal mass range (m/z 50-800). The concentration for the samples was 6.7 mM in a solution 1:1 MeOH-AcNH_4_ 45 mM


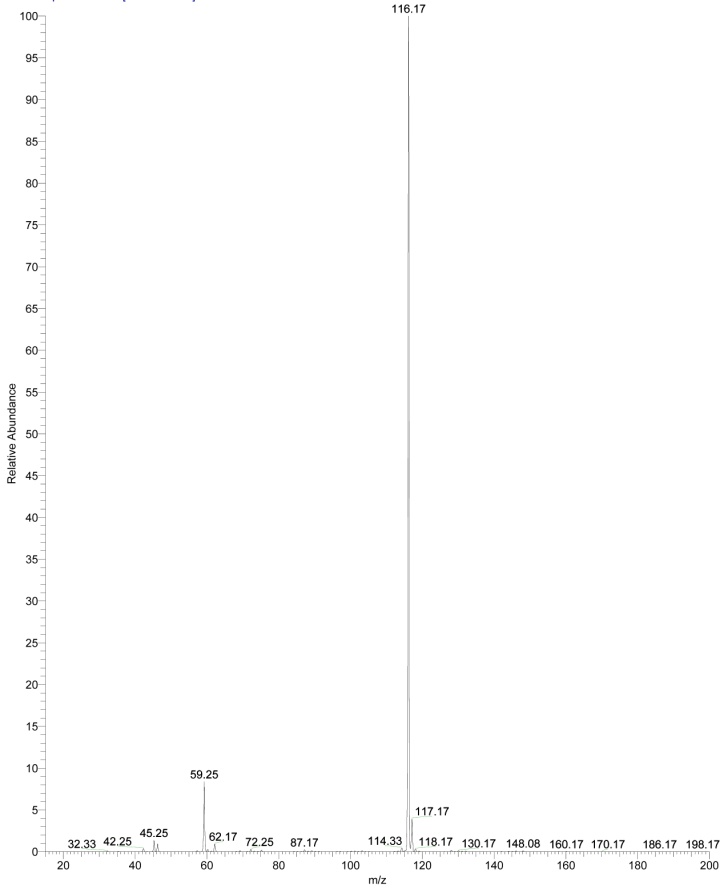


Mass spectrum of L-Val standard at low mass range (m/z 15-200), in negative ion mode. The concentration was 10mM in a solution 1:1 MeOH-AcNH_4_


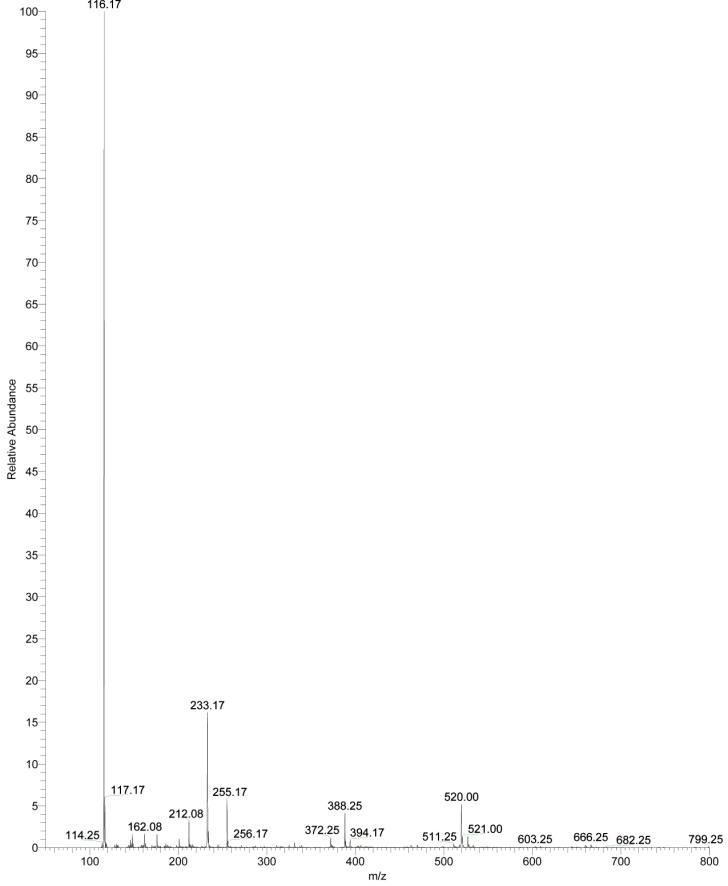


Mass spectrum of L-Val standard at normal mass range (m/z 50-800), in negative ion mode. The concentration was 10mM in a solution 1:1 MeOH-AcNH_4_


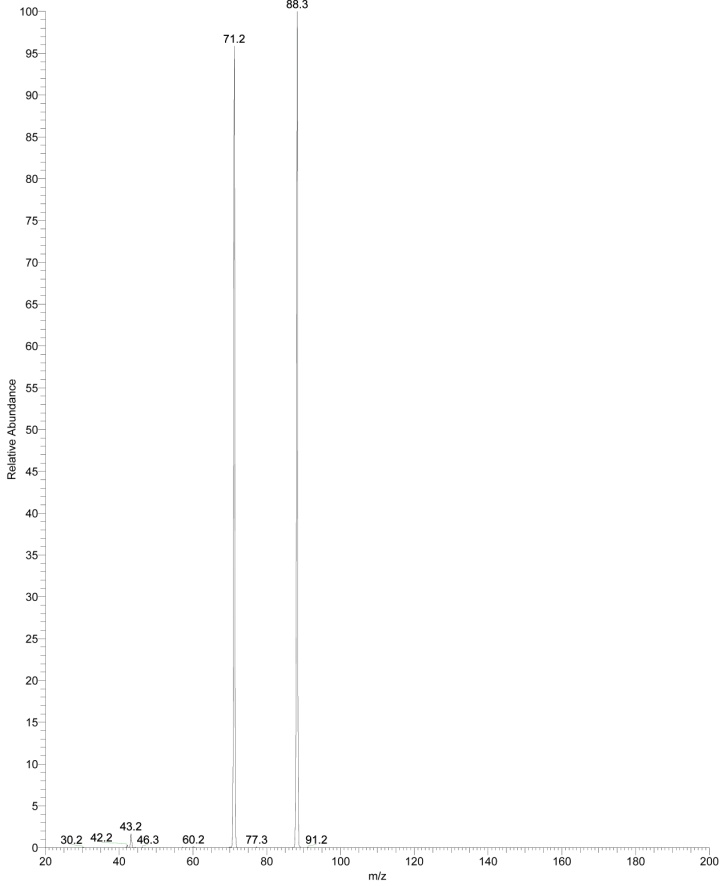


Fragmentation mass spectrum of the ion m/z 88 (2A) detected in positive ion mode, low mass range, for L-Leu irradiated in vacuum condition


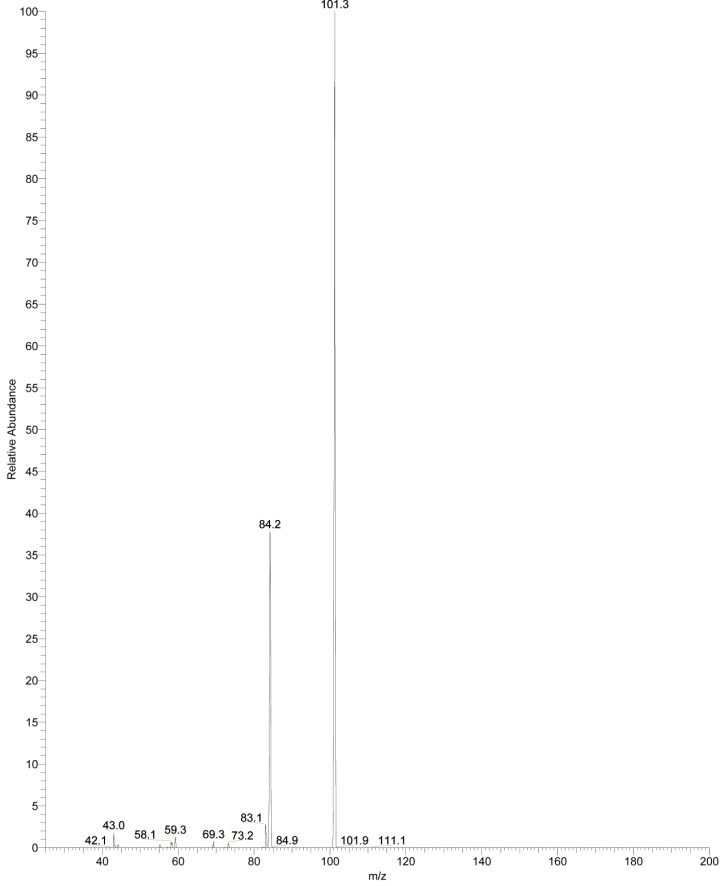


Fragmentation mass spectrum of the ion m/z 101 (2B) detected in positive ion mode, low mass range, for L-Leu irradiated in vacuum condition


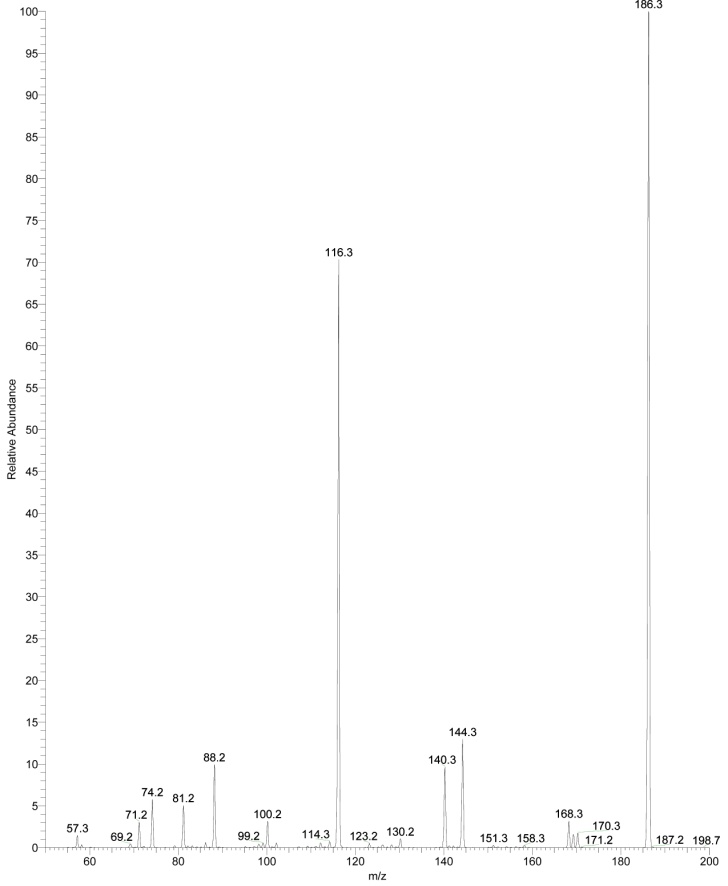


Fragmentation mass spectrum of the ion m/z 186 (2C) detected in positive ion mode, normal mass range, for L-Leu irradiated in vacuum condition


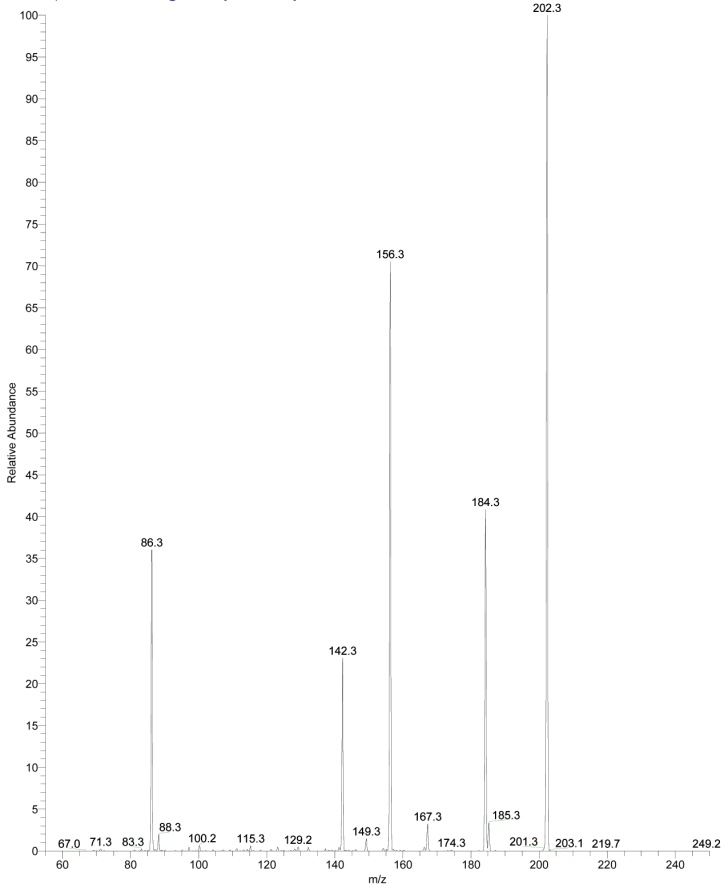


Fragmentation mass spectrum of the ion m/z 202 (2D) detected in positive ion mode, normal mass range, for L-Leu irradiated in vacuum condition


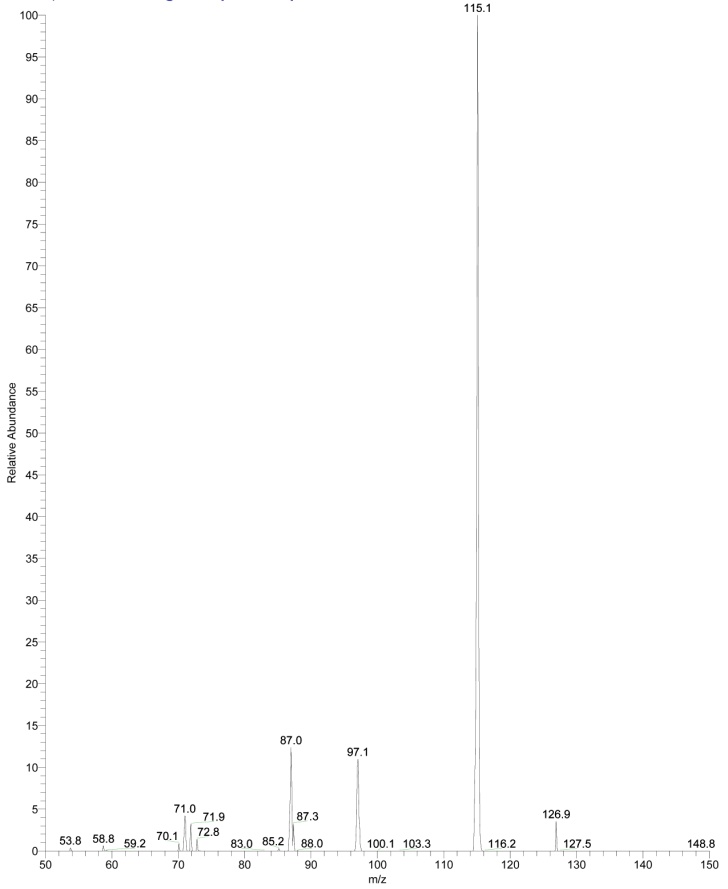


Fragmentation mass spectrum of the ion m/z 115 (2E) detected in negative ion mode, normal mass range, for L-Leu irradiated in vacuum condition


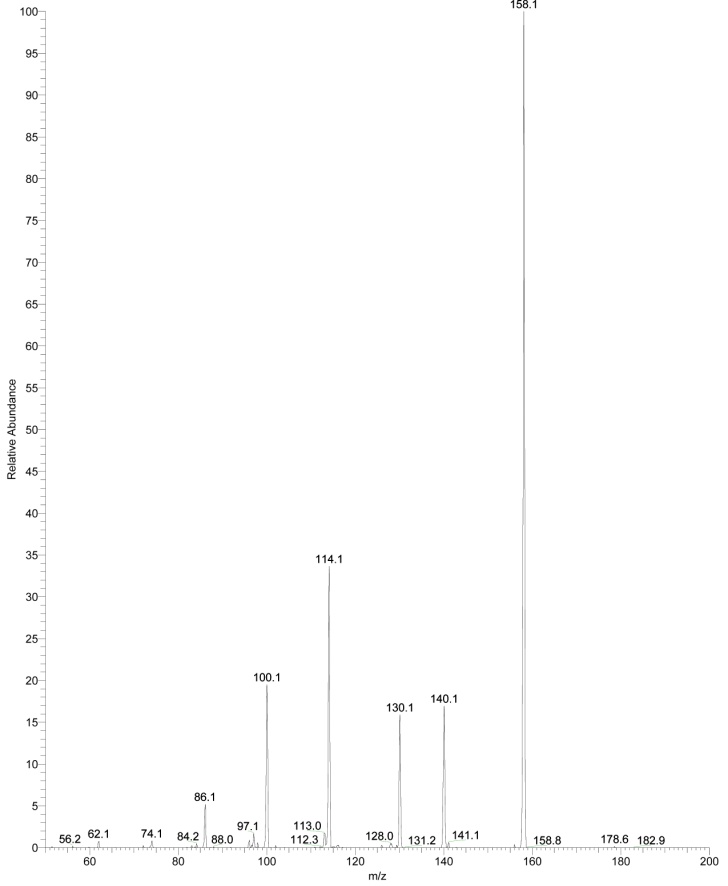


Fragmentation mass spectrum of the ion m/z 158 (2F) detected in negative ion mode, normal mass range, for L-Leu irradiated in vacuum condition


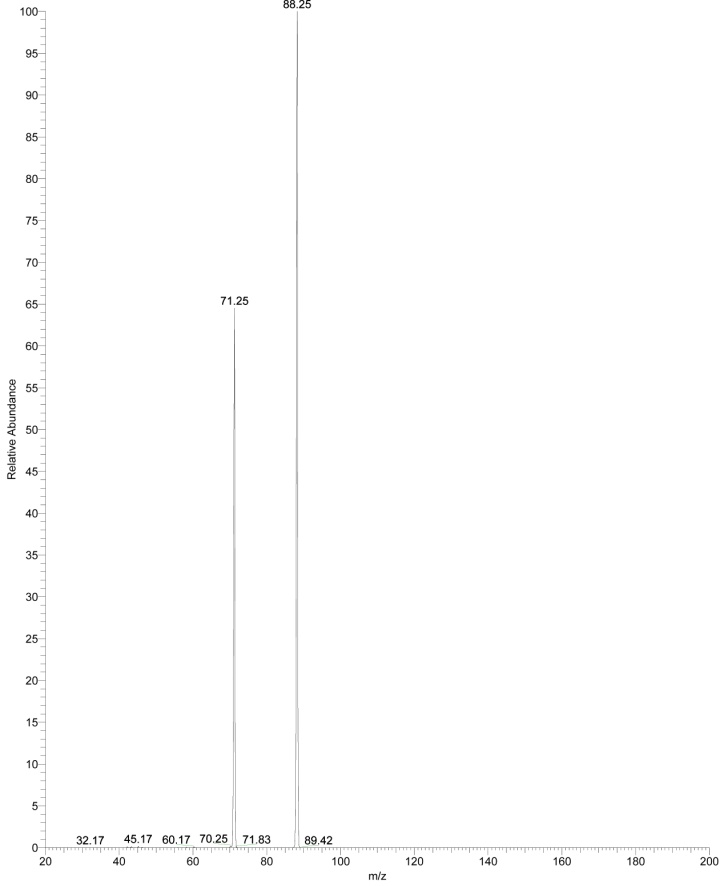


Fragmentation mass spectrum of the ion m/z 88 (4A) detected in positive ion mode, low mass range, for L-Ile irradiated in vacuum condition


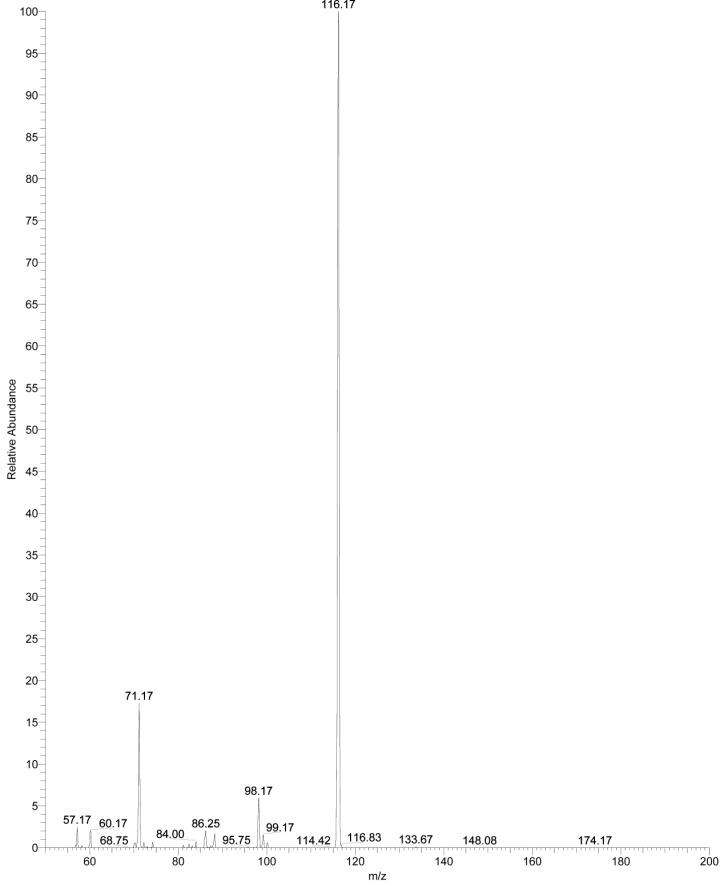


Fragmentation mass spectrum of the ion m/z 101 (4B) detected in positive ion mode, low mass range, for L-Ile irradiated in vacuum condition


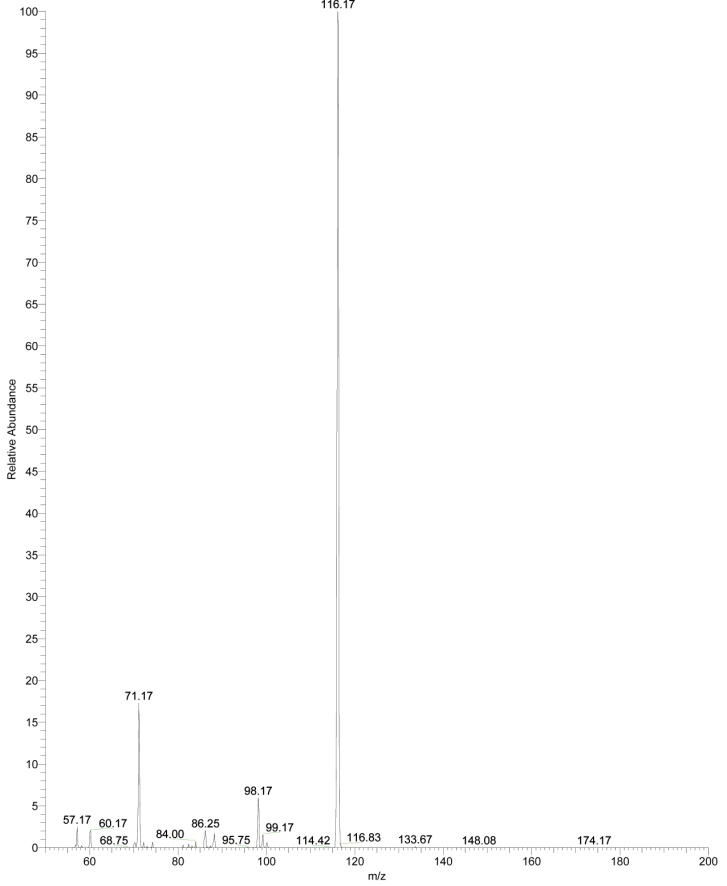


Fragmentation mass spectrum of the ion m/z 116 (4C) detected in positive ion mode, low and normal mass range, for L-Ile irradiated in vacuum condition


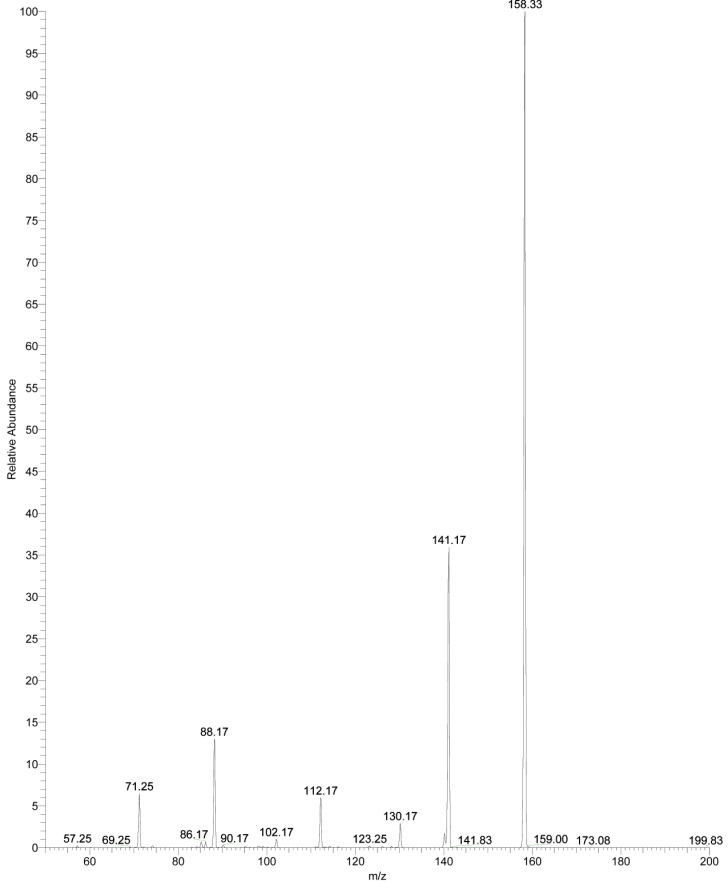


Fragmentation mass spectrum of the ion m/z 158 (4D) detected in negative ion mode, normal mass range, for L-Ile irradiated in vacuum condition


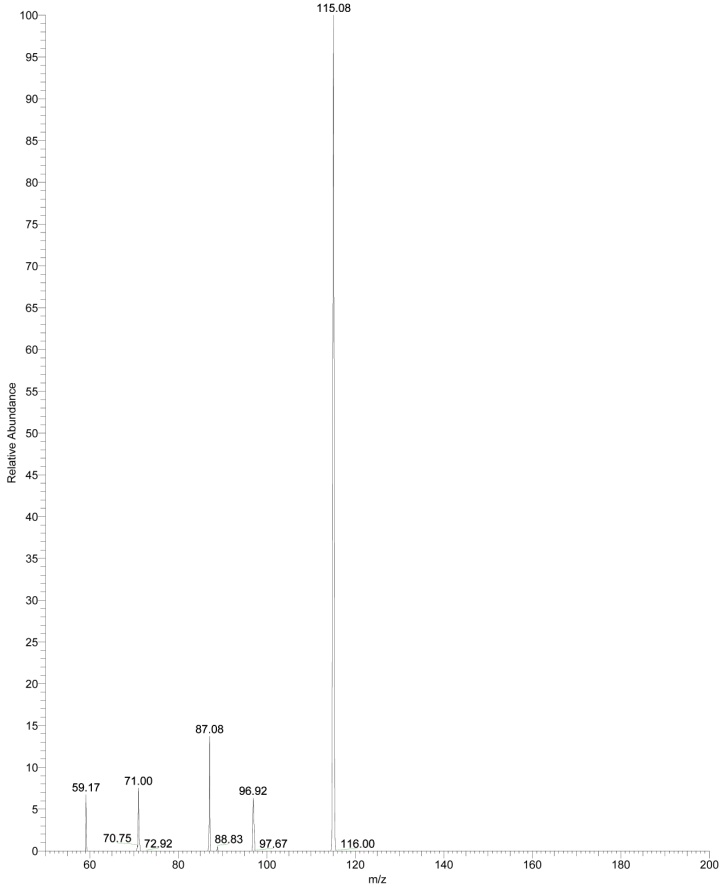


Fragmentation mass spectrum of the ion m/z 115 (4F) detected in negative ion mode, normal mass range, for L-Ile irradiated in vacuum condition


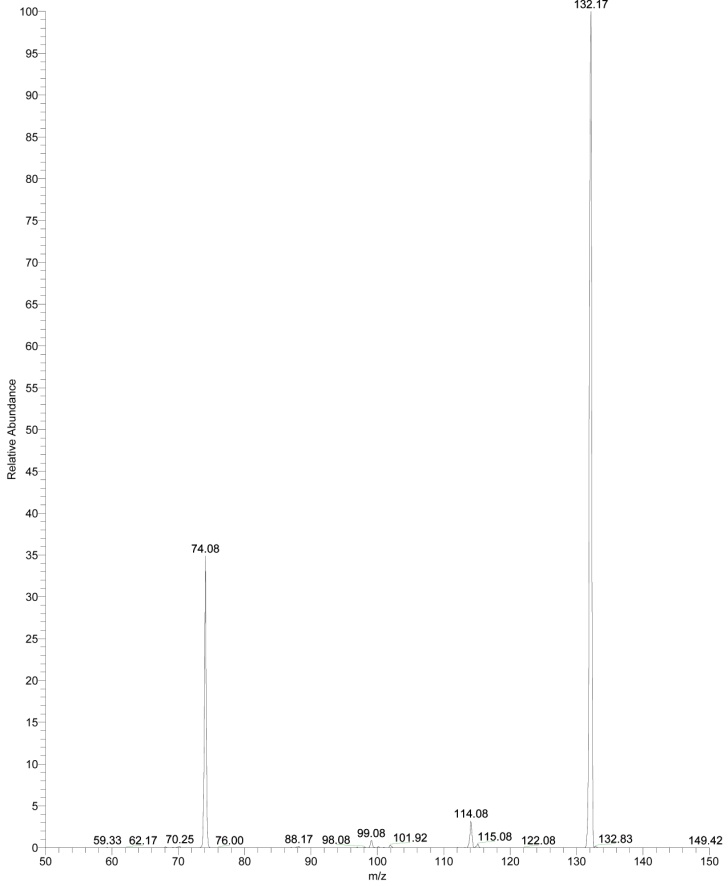


Fragmentation mass spectrum of the ion m/z 132 (6A) detected in negative ion mode, normal mass range, for L-Val irradiated in vacuum condition


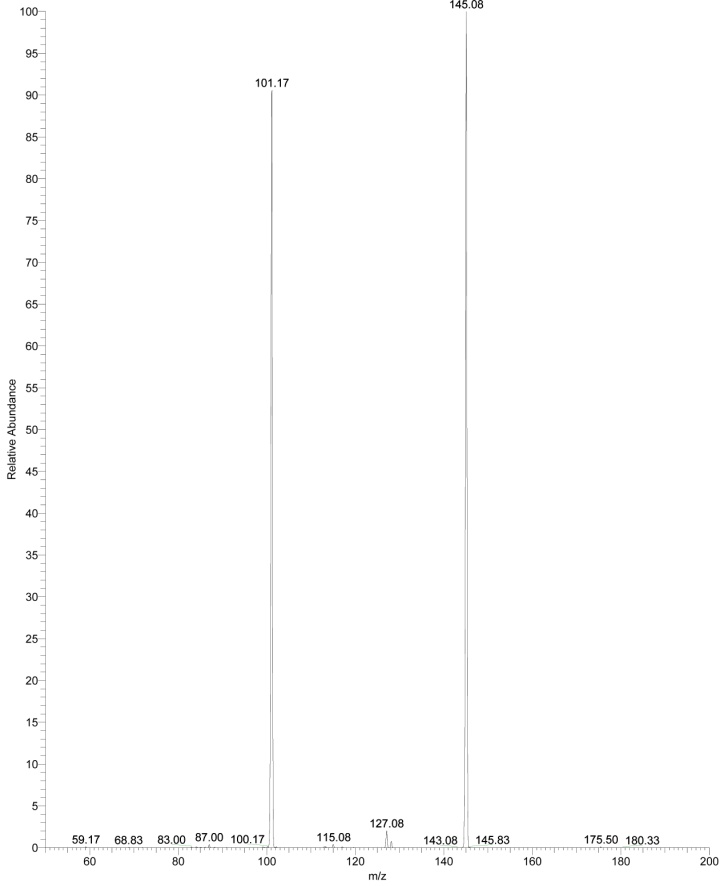


Fragmentation mass spectrum of the ion m/z 145 (6B) detected in negative ion mode, normal mass range, for L-Val irradiated in vacuum condition


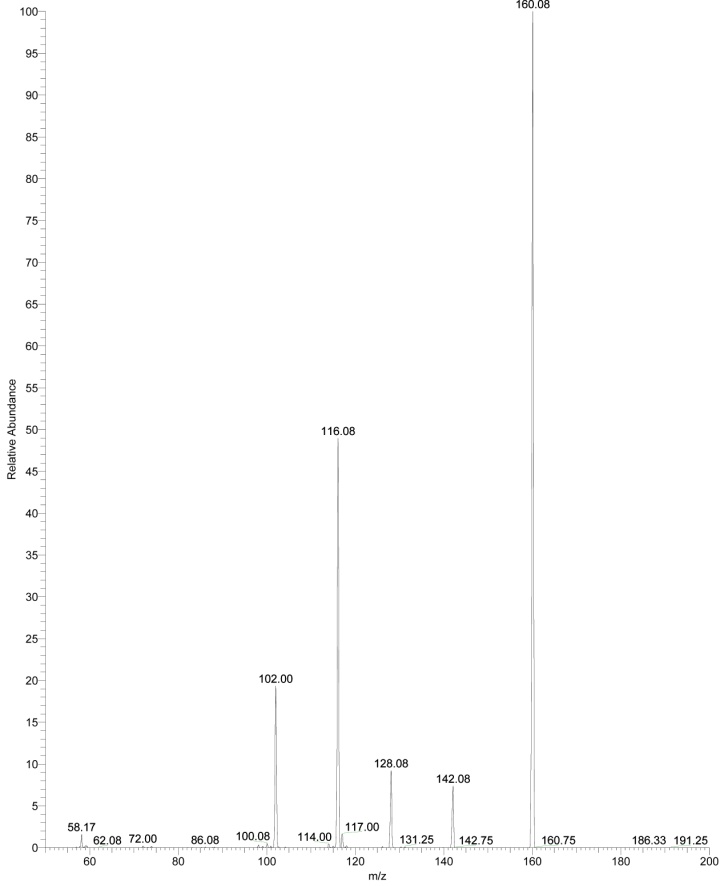


Fragmentation mass spectrum of the ion m/z 160 (6C) detected in negative ion mode, normal mass range, for L-Val irradiated in vacuum condition


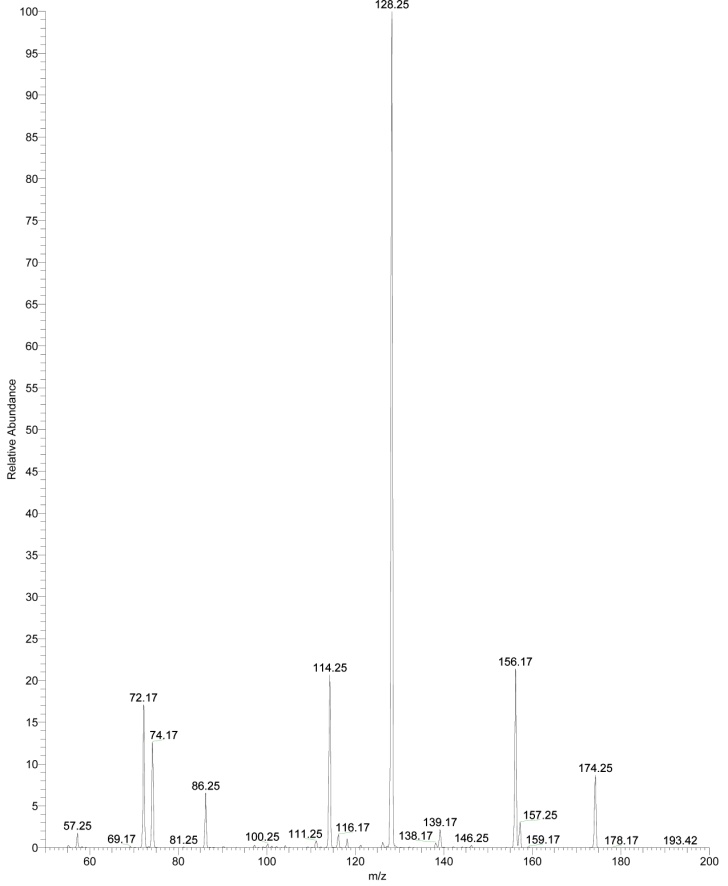


Fragmentation mass spectrum of the ion m/z 174 (6D) detected in positive ion mode, normal mass range, for L-Val irradiated in vacuum condition


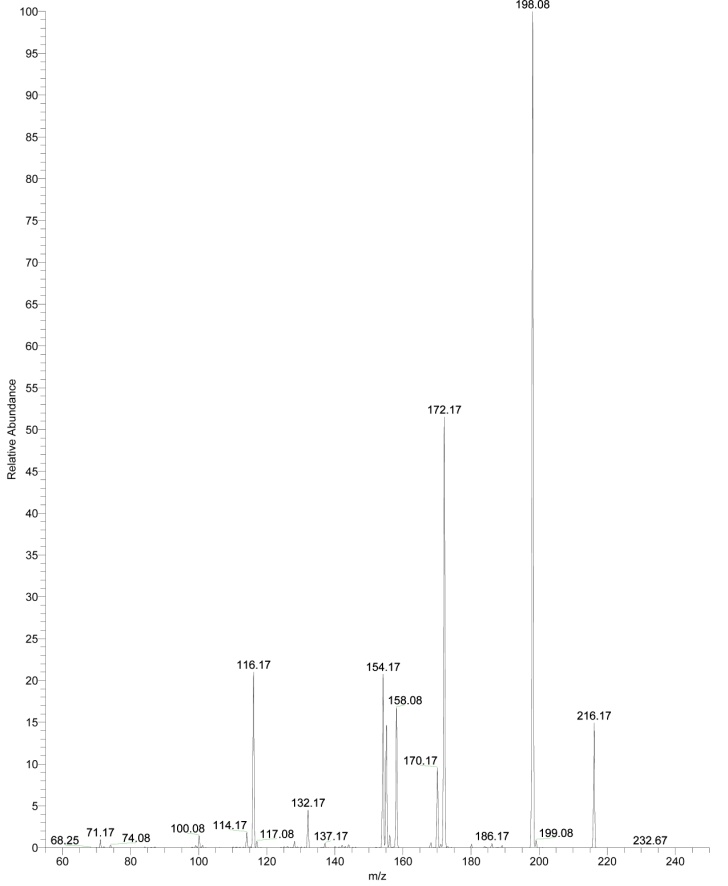


Fragmentation mass spectrum of the ion m/z 216 (6E) detected in negative ion mode, normal mass range, for L-Val irradiated in vacuum condition


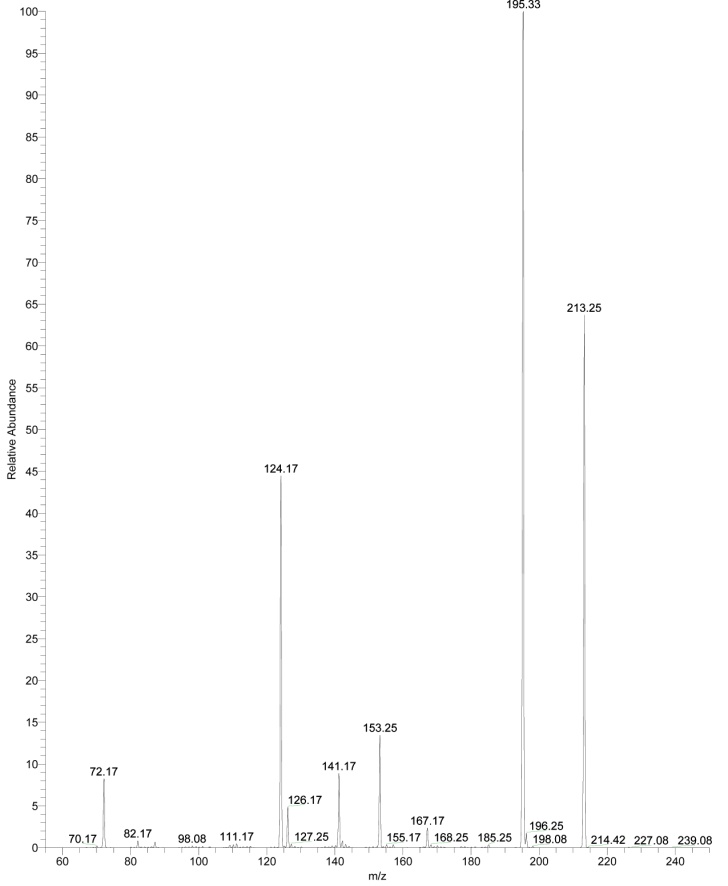


Fragmentation mass spectrum of the ion m/z 213 (6F) detected in positive ion mode, normal mass range, for L-Val irradiated in vacuum condition


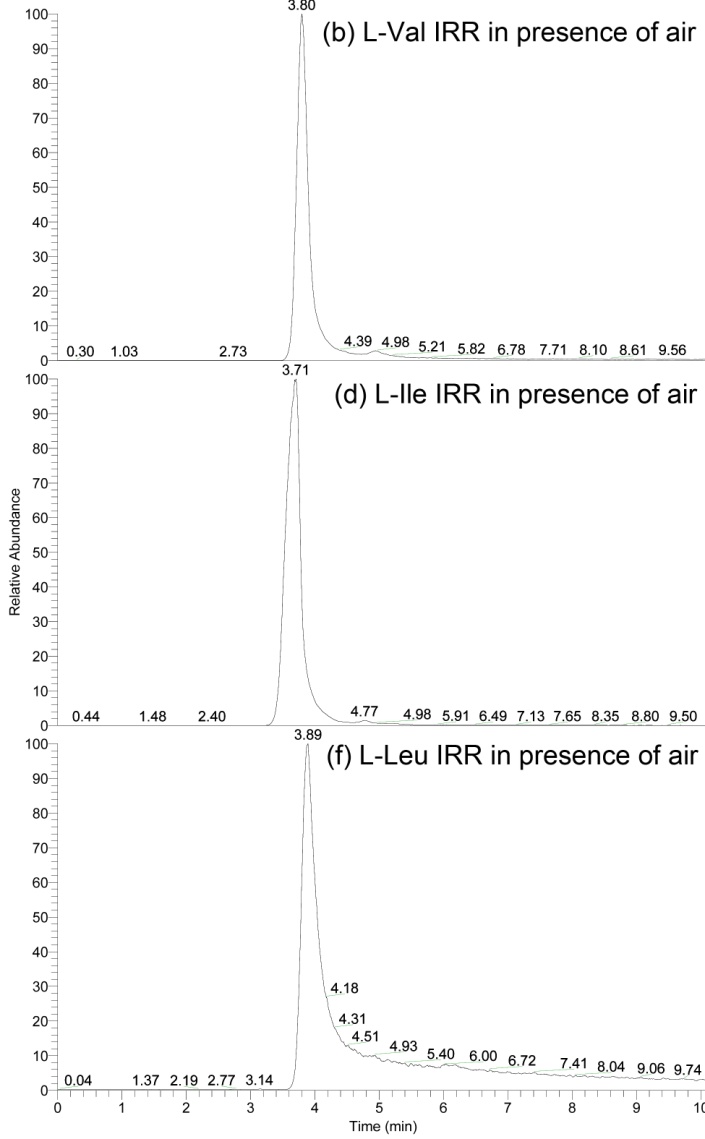

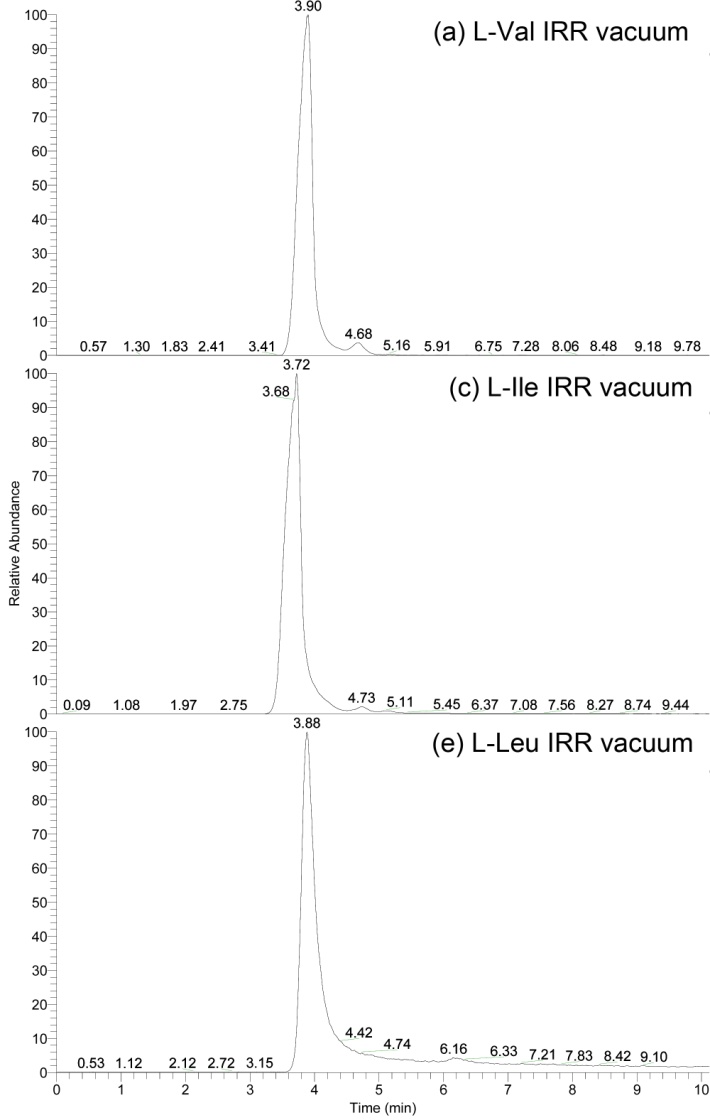


Chromatograms of L-Val, L-Ile and L-Leu irradiated in vacuum condition (a, c, e) and in presence of air (b, d, f)
